# Palmitoylation of the pore-forming subunit of Ca(v)1.2 controls channel voltage sensitivity and calcium transients in cardiac myocytes

**DOI:** 10.1073/pnas.2207887120

**Published:** 2023-02-06

**Authors:** Chien-Wen S. Kuo, Sara Dobi, Caglar Gök, Ana Da Silva Costa, Alice Main, Olivia Robertson-Gray, Daniel Baptista-Hon, Krzysztof J. Wypijewski, Hannah Costello, Tim G. Hales, Niall MacQuaide, Godfrey L. Smith, William Fuller

**Affiliations:** ^a^School of Cardiovascular and Metabolic Health, University of Glasgow, Glasgow G12 8QQ, UK; ^b^Division of Systems Medicine, Institute of Academic Anaesthesia, School of Medicine, University of Dundee, Ninewells Hospital and Medical School, Dundee DD1 9SY, UK; ^c^Center for Biomedicine and Innovations, Faculty of Medicine, Macau University of Science and Technology, Macau SAR, 999078 China

**Keywords:** heart, ion transport, acylation, excitation–contraction coupling

## Abstract

Mammalian voltage-activated L-type Ca^2+^ channels, such as Ca(v)1.2, control transmembrane Ca^2+^ fluxes in numerous excitable tissues. Here, we report that the pore-forming α1C subunit of Ca(v)1.2 is reversibly palmitoylated in rat, rabbit, and human ventricular myocytes. We map the palmitoylation sites to two regions of the channel: The N terminus and the linker between domains I and II. Whole-cell voltage clamping revealed a rightward shift of the Ca(v)1.2 current–voltage relationship when α1C was not palmitoylated. To examine function, we expressed dihydropyridine-resistant α1C in human induced pluripotent stem cell-derived cardiomyocytes and measured Ca^2+^ transients in the presence of nifedipine to block the endogenous channels. The transients generated by unpalmitoylatable channels displayed a similar activation time course but significantly reduced amplitude compared to those generated by wild-type channels. We thus conclude that palmitoylation controls the voltage sensitivity of Ca(v)1.2. Given that the identified Ca(v)1.2 palmitoylation sites are also conserved in most Ca(v)1 isoforms, we propose that palmitoylation of the pore-forming α1C subunit provides a means to regulate the voltage sensitivity of voltage-activated Ca^2+^ channels in excitable cells.

## Significance

Proper functioning of the voltage-dependent Ca^2+^ channel Ca(v)1.2 is indispensable for the normal physiology of smooth, skeletal, and cardiac muscles and for that of neurons and endocrine cells. Here, we report that the pore-forming α1C subunit of Ca(v)1.2 is palmitoylated in cardiac tissue and that this modification controls the voltage sensitivity of Ca(v)1.2 in voltage-clamped engineered cell lines. Ca^2+^ transients in human induced pluripotent stem cell–derived cardiomyocytes expressing unpalmitoylatable α1C are significantly smaller compared to those in cells expressing wild-type subunits. Targeting the shift in Ca(v)1.2 voltage activation properties caused by α1C palmitoylation could reduce susceptibility to fatal cardiac arrhythmias. Hence, we provide a mechanistic insight into the regulation of this physiologically important cardiac ion channel.

Voltage-activated channels facilitate the movement of ions across the membranes of excitable tissues in response to changes in membrane potential. The L-type Ca^2+^ channel mediates the depolarization-induced entry of Ca^2+^ into numerous cell types, thereby controlling excitation–contraction coupling in smooth and striated muscles, excitation–secretion coupling in endocrine cells, and neurotransmitter release in neurons (1). The precise control of L-type Ca^2+^ channel activity is therefore important in a diverse range of physiological settings—from the contraction of cardiac muscle in the control of cardiac output to the contraction of blood vessels in the control of blood pressure and the secretion of insulin in glucose homeostasis.

Voltage-activated Ca^2+^ channels are composed of multiple subunits, including a pore-forming α subunit and an accessory β subunit (2). The α subunit (α1C in the cardiac L-type Ca^2+^ channel Ca(v)1.2) is a transmembrane protein that has four domains (I to IV, each with six membrane-spanning units), which are connected by intracellular loops. The β subunit is cytosolic, interacts with the intracellular loop between domains I and II of the α subunit, and regulates numerous aspects of channel behavior (3).

There are four subfamilies of β subunits (β1 to β4) encoded by distinct genes which can be alternatively spliced (4). Specifically, β subunits enhance Ca(v)1.2-mediated currents by promoting channel exit from the endoplasmic reticulum (4). Once Ca(v)1.2 is at the cell surface, β subunits promote channel activation by shifting the voltage dependence of opening to more hyperpolarized potentials and accelerating channel activation (5, 6). Two forms of activity-dependent inactivation control most voltage-sensitive Ca^2+^ channels: Voltage-dependent inactivation (VDI) and calcium-dependent inactivation (CDI). Both VDI and CDI contribute to regulation of neuronal P/Q-type (Ca(v)2.1) Ca^2+^ channels, (7, 8) whereas CDI usually dominates for L-type channels such as Ca(v)1.2 in the heart (8–10). Most β subunits enhance both VDI and CDI, but β2a specifically reduces VDI (4).

Numerous regulatory pathways control Ca(v)1.2 activity. Its activation by protein kinases, which was previously thought to involve direct α1C phosphorylation, has recently been shown to involve the phosphorylation-mediated dissociation of the small G protein Rrad from the β subunit of Ca(v)1.2. (11) Palmitoylation, the reversible attachment of a fatty acid (usually palmitate) to cysteine thiols, is a posttranslational modification that regulates the activity of numerous ion channels and transporters. (12–21) Cysteines are usually palmitoylated based on their proximity to the membrane and hence the active site of the palmitoylating enzyme. No primary sequence features specifically directing palmitoylation of individual cysteines have been described, which makes palmitoylation site prediction haphazard. The regulatory β2a subunit of Ca(v)1.2 is palmitoylated shortly after being translated, which enhances the channel's activity (22, 23). However, the palmitoylation of the channel’s other subunits has yet to be investigated. In this study, we investigated the palmitoylation of the α1C subunit of Ca(v)1.2. We mapped the palmitoylation sites to the channel’s amino terminus and I–II linker and found a shift in the voltage dependence of activation in unpalmitoylatable Ca(v)1.2 channels compared to wild-type channels. In induced pluripotent stem cell-derived cardiac myocytes (CMs) expressing unpalmitoylatable α1C subunits, Ca^2+^ transient amplitudes were significantly reduced compared to wild type (WT). We conclude that α1C palmitoylation regulates channel properties of profound importance for normal L-type Ca^2+^ channel activity and for cardiac function.

## Results

### Ca(v)1.2 α1C Subunit Is Palmitoylated in Ventricular Tissue.

**Fig. 1.**
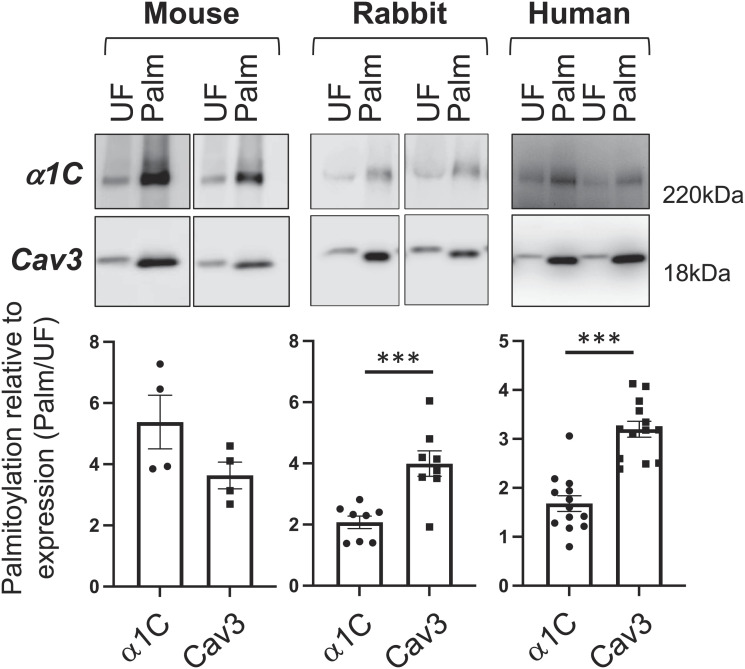
Ca(v) 1.2 α1C subunit is palmitoylated in mouse, rabbit, and human ventricular tissues. Palmitoylated proteins were purified by resin-assisted capture of acylated proteins (acyl-RAC) and immunoblotted as shown. The bar chart below each blot indicates the abundance of Ca(v)1.2 α1C and caveolin 3 (Cav3) in the purified palmitoylated fraction (Palm) relative to the corresponding unfractionated lysate (UF). N = 4 (mouse), N = 8 (rabbit), N = 7 (human). ***P < 0.001, unpaired *t* test. Error bars represent SEM.

We began by identifying the Ca(v)1.2 α1C subunit in a proteomic screen of palmitoylated proteins from rat ventricular myocytes. We next investigated its palmitoylation. Palmitoylated proteins were purified using resin-assisted capture of acylated proteins [acyl resin-assisted capture (acyl-RAC)] from mouse, rabbit, and human ventricular tissues (Fig. 1) (24). This technique detects the presence of a thioester bond on cysteine side chains under strongly denaturing conditions. Only proteins with a fatty acid esterified to a cysteine (and not those interacting with them) are purified. We estimated the fraction of palmitoylated Ca(v)1.2 α1C subunits in cardiac muscle by comparing their enrichment following acyl-RAC with the enrichment of the stoichiometrically palmitoylated protein, caveolin 3 (12). In large mammals (rabbits and humans), α1C was ~60% palmitoylated, significantly less than caveolin 3. In mice, α1C was close to stoichiometrically palmitoylated (Fig. 1).

### Palmitoylation Is Not Required to Direct α1C to Cholesterol-Rich Caveolar Microdomains.

**Fig. 2.**
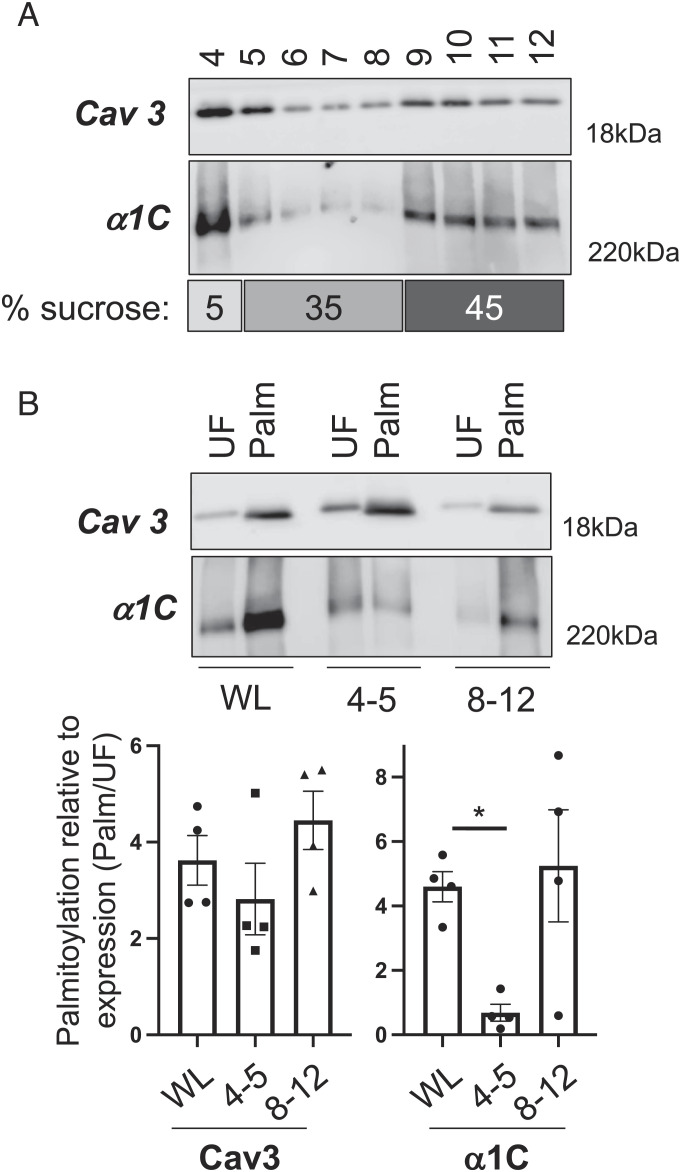
Palmitoylation and subcellular localization of the Ca(v)1.2 α1C subunit. (*A*) Western blot of caveolae prepared from mouse ventricular myocytes using a standard discontinuous sucrose gradient (sucrose concentrations in the numbered gradient fractions are indicated under the blots). Caveolin-enriched membranes were harvested from gradient fractions 4 and 5. (*B*) Palmitoylated proteins were prepared from whole cardiac lysates (WL) and pooled buoyant caveolar (4+5) and dense noncaveolar (8−12) fractions. The bar charts show the amount of Ca(v)1.2 α1C and caveolin 3 (Cav3) in the purified palmitoylated fraction (Palm) relative to the corresponding unfractionated whole lysate/pooled gradient fractions (UF). Caveolin 3 is equally palmitoylated in all fractions, but little Ca(v)1.2 α1C present in buoyant caveolar membranes is less palmitoylated. *P < 0.05 compared to WL, ANOVA followed by Dunnett’s multiple comparisons test, N = 4.

Palmitoylation is proposed to partition proteins to detergent-resistant membranes, such as caveolae, (25) so we investigated the localization of palmitoylated Ca(v)1.2 to cardiac caveolae. A small proportion of Ca(v)1.2 α1C subunit localizes to buoyant caveolin-enriched microdomains prepared from mouse ventricular tissue using a standard discontinuous sucrose gradient (Fig. 2*A*). Buoyant (fractions 4 and 5) and dense (pooled fractions 8 to 12) membranes were used in acyl-RAC experiments to purify palmitoylated proteins (Fig. 2*B*). We found palmitoylated α1C subunits present in both buoyant and dense membranes. Surprisingly, the small fraction of α1C localized in buoyant membranes was significantly less palmitoylated than that of α1C from an unfractionated ventricular lysate (Fig. 2*B*), which indicates that α1C palmitoylation is not required for the localization of Ca(v)1.2 to cardiac caveolae.

### α1C Is Palmitoylated at Multiple Intracellular Cysteines.

**Fig. 3.**
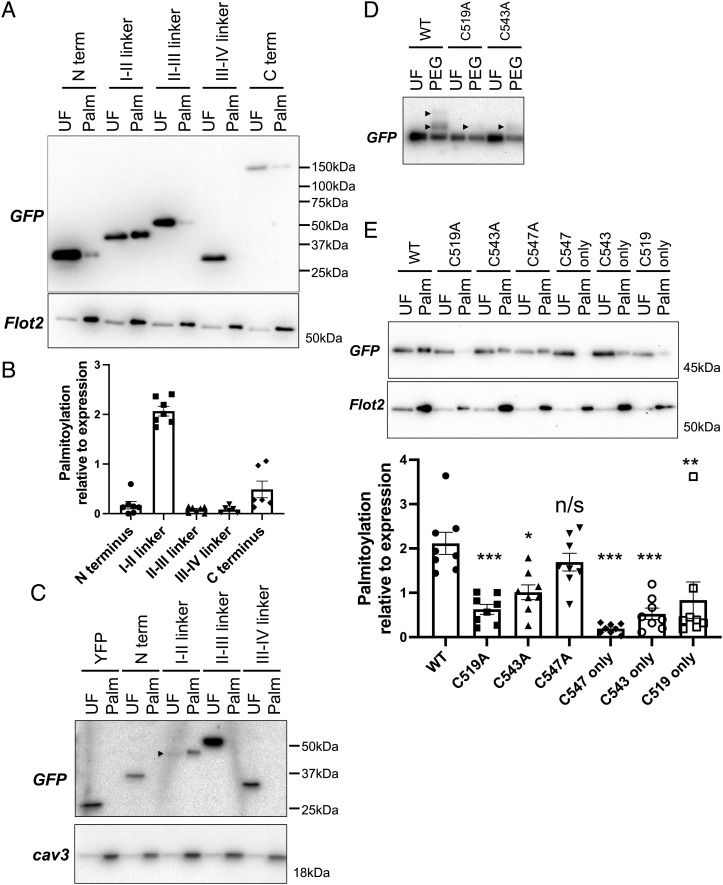
Palmitoylation of the I–II linker of α1C in transfected HEK cells. (*A*) YFP fusion proteins of the α1C intracellular regions were expressed in HEK cells, and palmitoylated proteins (Palm) were then purified and immunoblotted alongside unfractionated (UF) cell lysates. The palmitoylated protein flotillin 2 is a positive control for the acyl-RAC reaction. (*B*) Palmitoylation of YFP fusion proteins normalized to expression in HEK cells (N = 9). (*C*) The I–II linker (but no other intracellular regions) is also palmitoylated in the cardiomyoblast H9C2 line. For clarity, the I–II linker is marked with an arrowhead. Blots are representative of two independent experiments. (*D*) PEGylation assay identifies dual palmitoylation of the I–II linker. In this assay, palmitates are replaced with a 5-kDa PEG molecule, which causes a band shift on sodium dodecyl sulfate–polyacrylamide gel electrophoresis (SDS-PAGE) (arrowhead), according to the number of palmitoylation sites. Mutation of either C519 or C543 removes one palmitoylation site. (*E*) Identification of the palmitoylated cysteines in a YFP fusion of α1C I–II linker. Single and double cysteine-to-alanine mutants were expressed in HEK cells, and palmitoylated proteins prepared by resin-assisted capture (WT, wild type; “only” refers to all cysteines, but this one removed). Acyl-RAC captures proteins regardless of whether they are singly or doubly palmitoylated. Capture of each mutant (relative to expression) is shown in the bar chart below the representative blot (error bars represent SEM). Mutation of a single cysteine does not abolish palmitoylation, but mutation of cysteines in positions 519 and 543 does (UF: unfractionated cell lysate, Palm: palmitoylated proteins, N = 8). Statistical comparisons were made using ANOVA followed by Dunnett’s multiple comparisons test and are to WT. ***P < 0.001, **P < 0.005, *P < 0.05, n/s, not significant.

To map α1C palmitoylation sites, we expressed yellow fluorescent protein (YFP) fusion proteins of the 5 intracellular regions (N and C termini, I–II, II–III, and III–IV linkers) of the human α1C subunit in HEK cells and measured their palmitoylation by acyl-RAC (Fig. 3*A*). The I–II linker is the region that undergoes the most quantitatively significant level of palmitoylation in α1C (Fig. 3*B*). In some experiments, a small fraction of the α1C intracellular carboxyl terminus was palmitoylated. We confirmed these findings in a second cell type, rat cardiomyoblast H9C2 cells (Fig. 3*C*). The I–II linker contains three cysteines (numbered 519, 543, and 547 in the rabbit splice variant CACH2A of α1C). We used acyl-polyethylene glycol (PEG) exchange, which generates a 5-kDa band shift for each palmitoylation site in a protein, to establish that two cysteines in this region are palmitoylated (Fig. 3*D*). Alanine mutagenesis indicates that C519 and C543 are both palmitoylated but that C547 is not (Fig. 3*E*). C519 is the principal palmitoylation site as its removal has the greatest impact on palmitoylation of the I–II linker.

**Fig. 4.**
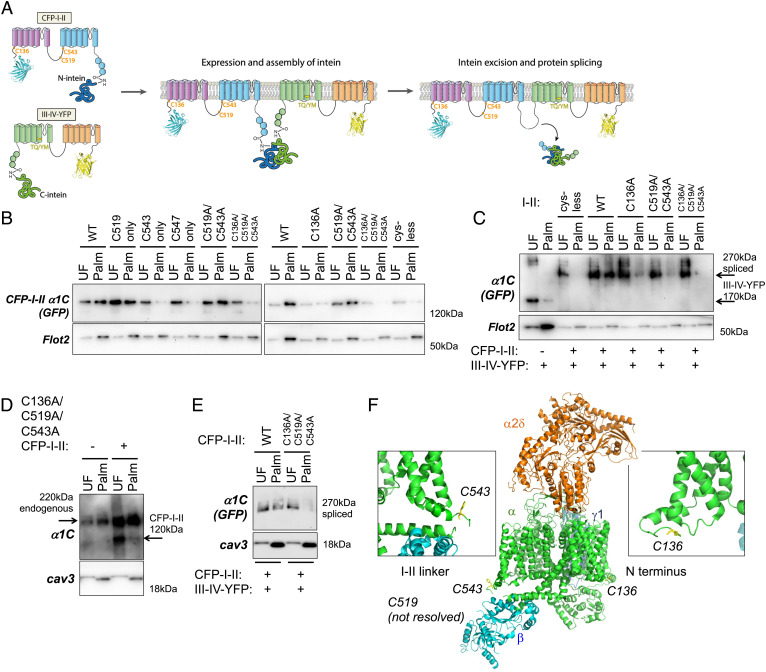
Expression and palmitoylation of α1C using the split intein system. (*A*) Intein splicing supports the assembly of mature, dihydropyridine-resistant α1C from CFP-tagged I-II and YFP-tagged III-IV. The positions of the palmitoylation sites and the mutations that render the channel nifedipine insensitive (TQ/YM) are marked. (*B*) Palmitoylation site mapping in α1C using CFP-tagged I-II fused to a split intein. Mutation of C519 and C543 in the I–II linker does not abolish α1C palmitoylation, but the additional mutation of C136 does. (*C*) Palmitoylation of intein-spliced channels in HEK cells. A spliced channel with all intracellular cysteines in the I–II half mutated (cys-less) is not palmitoylated. Mutagenesis confirms palmitoylation of C136, C519, and C543 in the spliced channel. (*D*) Adenoviral expression of C136A/C519A/C543A [I-II]-N-intein in rat ventricular myocytes confirms that the palmitoylation sites mapped in HEK cells are recapitulated in ventricular muscle (detected with anti-α1C). (*E*) No palmitoylation of spliced C136A/C519A/C543A α1C in rabbit ventricular myocytes (detected with anti-GFP). (*F*) Position of palmitoylated cysteines in Ca(v)1.1 structure from PDB entry 5GJW generated using PyMOL. The analogous residues to C136 (C33) and C543 (F421) are colored yellow. *Insets* show the same regions viewed from the other side of the protein.

To identify palmitoylation sites in full-length α1C, HEK cells were transfected with two separate plasmids: one expressing the cyan fluorescent protein (CFP)-tagged N-terminal half (CFP-I-II) and the other expressing the YFP-tagged C-terminal half (III-IV-YFP) of α1C fused to a split intein. We employed the split intein system because this would allow us to express α1C in myocytes using adenoviruses. Full-length α1C exceeds the packaging limit for adenoviruses, so it must be delivered using two separate vectors. When split between two polypeptides in the same cell, split inteins assemble to form an active intein that splices the two polypeptides together and excises the intein (Fig. 4*A*). This system is validated for expressing α1C in CMs (26). In the presence of the transmembrane (TM) domains, we found an additional palmitoylation site C136 in the α1C N terminus and C519 and C543 in the I–II linker (Fig. 4*B*). We coexpressed CFP-I-II with III-IV-YFP to generate intein-spliced channels in HEK cells (Fig. 4*C*). Combining III-IV-YFP with a CFP-I-II mutant with all intracellular cysteines mutated to alanine generates a nonpalmitoylated channel, confirming that all palmitoylation sites lie in the N-terminal half of the channel. Mutagenesis of the C136 or C519/C543 residues reduces, but does not abolish, the palmitoylation of the spliced channel. A spliced channel lacking all three palmitoylation sites is not palmitoylated (Fig. 4*C*).

We generated adenoviruses that encode split α1C and expressed wild-type and mutated α1C in ventricular myocytes isolated from rat and rabbit hearts. The mutation of C136, C519, and C543 in the N-terminal half of α1C abolished its palmitoylation in rat myocytes (Fig. 4*D*). The same palmitoylation pattern was observed in rabbit myocytes (Fig. 4*E*).

The positions of these palmitoylation sites in the structure of skeletal muscle Ca(v)1.1 (27) are shown in Fig. 4*F*. C136 and C543 lie close to the membrane, but no structure around C519 has as yet been resolved, which suggests that this region of the I–II linker is either unstructured or can adopt >1 structure, which may be influenced by palmitoylation at C519.

### Palmitoylation of α1C Modifies the Voltage Dependence of Ca(v)1.2 in Cultured Cells.

**Fig. 5.**
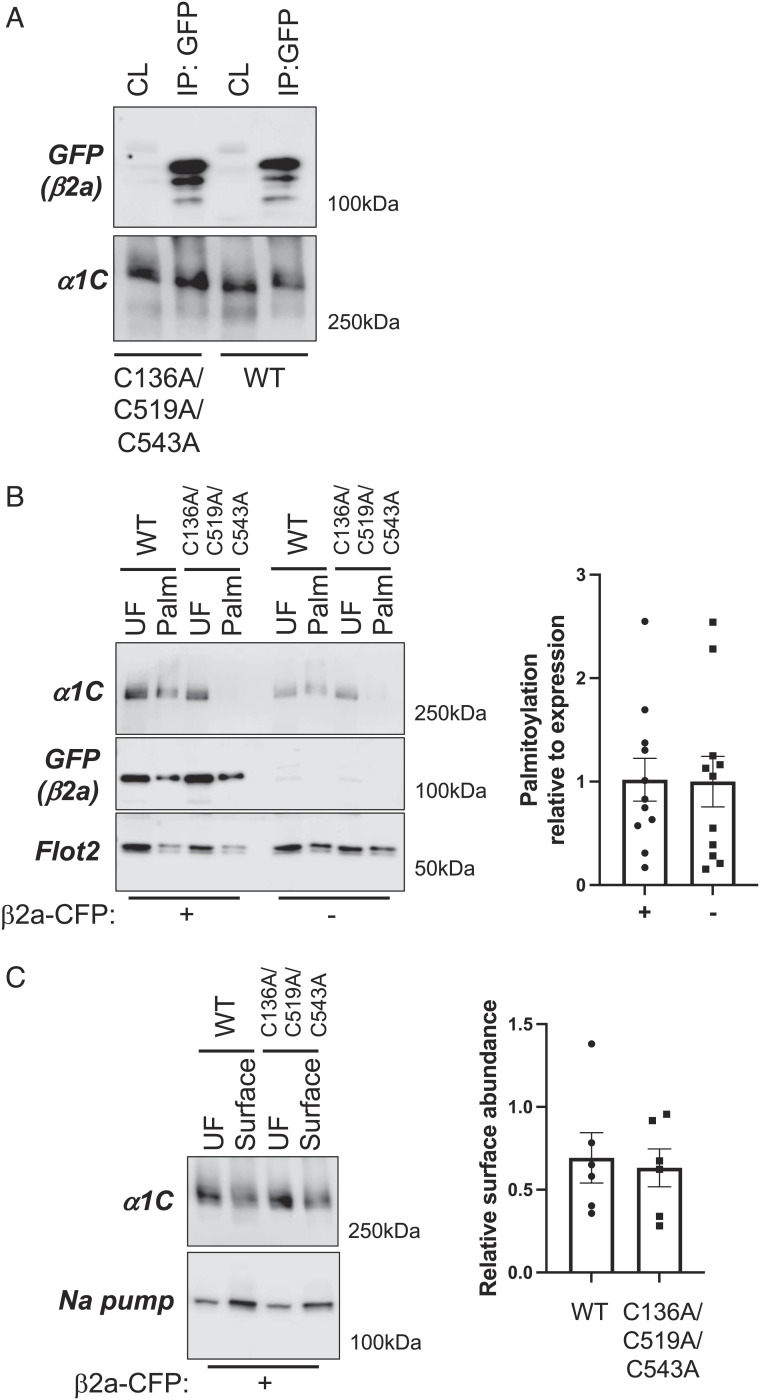
Impact of palmitoylation on α1C association with β2a and trafficking. (*A*) Western blot showing the coimmunoprecipitation of WT and unpalmitoylatable α1C with β2a-CFP from HEK cells stably expressing WT and unpalmitoylatable α1C transfected with β2a-CFP. CL: detergent-solubilized cell lysate, IP: immunoprecipitated proteins. (*B*) Palmitoylation assay from HEK cells stably expressing WT and unpalmitoylatable α1C transfected with β2a-CFP (*Left*, +) or in the absence of β2a-CFP (*Right*, −). UF: unfractionated cell lysate, Palm: palmitoylated proteins. The bar chart shows α1C palmitoylation relative to expression in the presence (+) and absence (−) of β2a-CFP (N = 11). (*C*) Impact of α1C palmitoylation on its steady-state expression at the plasma membrane in the presence of β2a-CFP. Surface membrane proteins were biotinylated, purified, and immunoblotted for α1C and the housekeeping protein Na pump. The bar chart shows α1C surface membrane abundance relative to expression (N = 6).

We investigated the functional impact of α1C palmitoylation using engineered Flp-In 293 T-Rex cell lines stably expressing tetracycline-inducible wild-type and nonpalmitoylated (C136A/C519A/C543A) α1C. To evaluate the impact of α1C palmitoylation on its interaction with Ca(v)1.2 β subunit, cells were transfected with β2a-CFP, and copurification of the two proteins was assessed by coimmunoprecipitating using anti-GFP. Equal quantities of wild-type and nonpalmitoylated α1C were copurified with β2a in this assay (Fig. 5*A*), indicating that the physical interaction between the two proteins is not modified by palmitoylation of the α1C I–II linker. Since the β2a is also palmitoylated, we investigated whether α1C palmitoylation was modified by coexpressing β2a and found it unchanged (Fig. 5*B*). Nor did palmitoylation influence the amount of α1C expressed at the cell surface in the presence of β2a (assessed using membrane-impermeable biotinylation reagents; Fig. 5*C*).

**Fig. 6.**
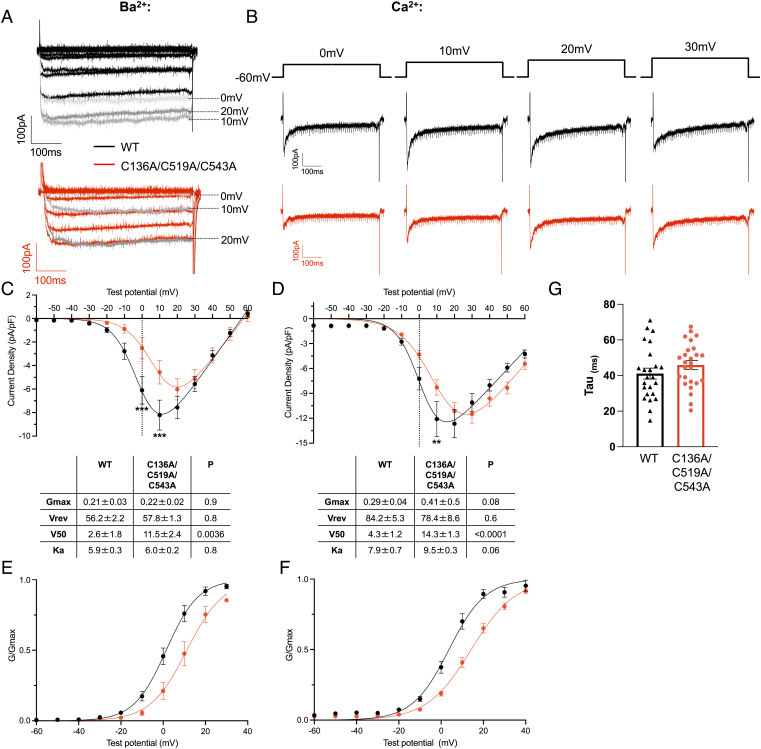
Impact of palmitoylation on Ca(v)1.2-mediated currents. HEK cells stably expressing wild-type or unpalmitoylatable α1C and transfected with β2a were voltage-clamped at −60 mV. 500-ms voltage steps from −60 mV to +60 mV at 10-mV increments were applied at 1 Hz. Representative examples of currents from wild-type (black) or unpalmitoylatable (red) channels with either Ba^2+^ (*A*) or Ca^2+^ (*B*) as the charge carrier are shown. Prominent inactivation was noted with Ca^2+^ as the charge carrier. Peak amplitudes from the families of currents shown in *A* and *B* were normalized to cell capacitance and plotted against the step voltage to construct the current–voltage relationships for experiments conducted with Ba^2+^ (C; wild-type N = 14, unpalmitoylatable N = 12) or Ca^2+^ (*D*; wild-type N = 27, unpalmitoylatable N = 24) as the charge carrier. Current densities at each step voltage were compared between wild-type and unpalmitoylatable channels; **P < 0.01, ***P < 0.001 (unpaired *t* test with Holm–Sidak correction for multiple comparisons). A Boltzmann function was fitted to the I–V relationships (*Methods*) to yield Gmax, Vrev, V50, and Ka parameters. The tables provide the parameters derived from individual cells, with *P* values obtained from unpaired *t* tests. G–V curves were drawn by calculating G/Gmax (using parameters obtained from the Boltzmann fits of I–V curves from individual cells, see *Methods*) for Ca(v)1.2 composed of wild-type or unpalmitoylatable α1C with Ba^2+^ (*E*) or Ca^2+^ (*F*) as the charge carrier. (*G*) Time constant for current decay for Ca(v)1.2 at +20 mV for wild-type (N = 24) and unpalmitoylatable (N = 26) α1C.

To evaluate the impact of α1C palmitoylation on Ca(v)1.2-mediated currents, we used whole-cell voltage clamping following tetracycline induction of α1C expression in cells transfected with β2a using either Ba^2+^ (Fig. 6 *A*, *C*, and *E*) or Ca^2+^ (Fig. 6 *B*, *D*, *F*, and *G*) as the charge carrier. For both Ba^2+^ (Fig. 6*C*) and Ca^2+^ (Fig. 6*D*) currents, we observed a rightward shift of the current–voltage (I–V) relationship for Ca(v)1.2 when α1C was nonpalmitoylated, such that currents in cells expressing WT α1C were significantly larger at 0 mV (Ba^2+^ currents) and +10 mV (Ba^2+^ and Ca^2+^ currents). Boltzmann fits of these recordings identified an ~10-mV positive shift of mean half-activation voltage (V50) when α1C was nonpalmitoylated. Conductance–voltage (G–V) curves identified the same shift in Ca(v)1.2 voltage dependence of activation but no significant change in slope factor (K_a_) using both charge carriers (Fig. 6 *E* and *F*).

We detected essentially no inactivation of Ba^2+^ currents (Fig. 6*A*), suggesting VDI is negligible in our experiments. Prominent inactivation was noted with Ca^2+^ as the charge carrier (Fig. 6*B*). Peak Ca^2+^ currents occurred at +20 mV for WT and +30 mV for nonpalmitoylated α1C. We assessed Ca(v)1.2 inactivation properties at +20 mV, a voltage at which Ca^2+^ entry was not different between the two groups. The inactivation time constant was not different between WT and nonpalmitoylated α1C (Fig. 6*G*), suggesting CDI of Ca(v)1.2 is not influenced by α1C palmitoylation.

### α1C Palmitoylation Controls Ca^2+^ Transient Amplitude in iPSC-Derived CMs.

**Fig. 7.**
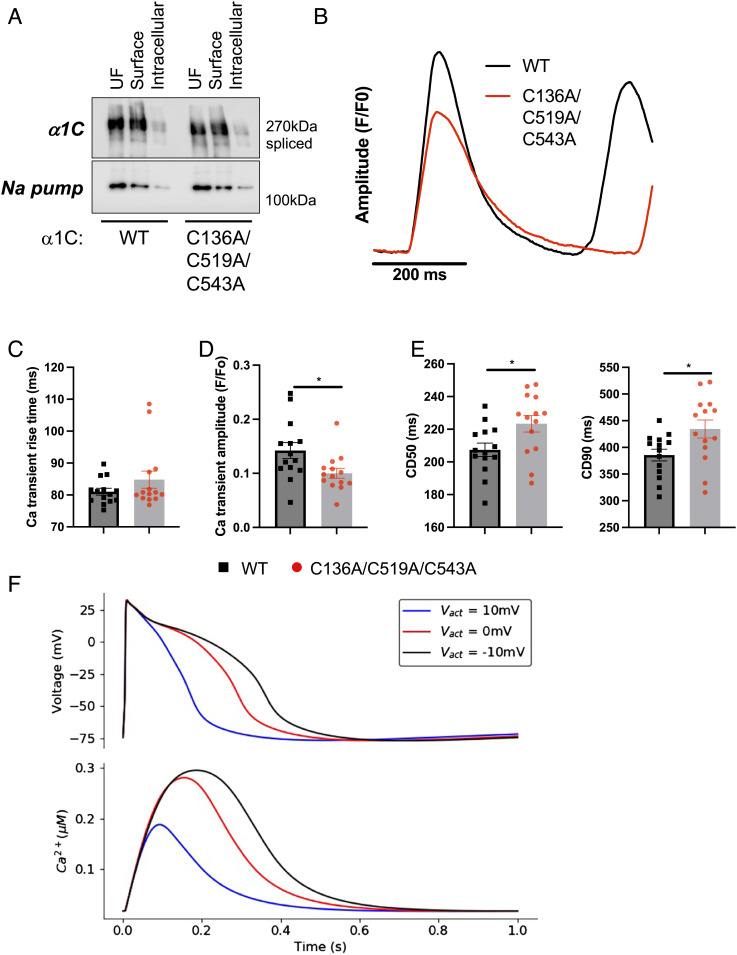
Ca^2+^ transient characteristics in iPSC-derived myocytes expressing wild-type and unpalmitoylatable α1C. (*A*) Impact of α1C palmitoylation on its steady-state expression at the plasma membrane in iPSC-derived myocytes. Surface membrane proteins were biotinylated, purified, and immunoblotted for α1C and the housekeeping protein Na pump. Representative of 3 independent experiments. (*B*) Ca^2+^ transients were measured from monolayers of iPSC-derived cardiomyocytes loaded with Fura2 by incubation with the -AM form and excited in an alternate fashion at 340 nm and 380 nm. Ca^2+^ transients were derived from the fluorescence ratio (F340/F380) and were analyzed using CellOPTIQ analysis software. Averaged Ca^2+^ transients indicate reduced Ca^2+^ transient amplitude and significant prolongation in the presence of unpalmitoylatable α1C compared to WT. (*C*) Ca^2+^ transient rise time is not influenced by α1C palmitoylation (N = 14). (*D*) Ca^2+^ transient amplitude is reduced in cells expressing unpalmitoylatable α1C (N = 14). (*E*) Ca^2+^ transient duration is significantly prolonged in the presence of unpalmitoylatable α1C. CD50: transient duration at 50% amplitude. CD90: transient duration at 90% amplitude. *P < 0.05 (unpaired *t* test) compared to WT, N = 14. Error bars represent SEM. (*F*) Modeled effects of altered V50 values for the L-type Ca^2+^ channel on the action potential and Ca^2+^ transient in human iPSC-derived cardiomyocytes. The V50 (V*_act_*) values were varied from the default value (−10 mV, black) to 0 mV (red) and +10 mV (blue).

We used adenoviruses that express a dihydropyridine-resistant split form of α1C (CFP-I-II and YFP-III-IV T1066Y/Q1070M) to investigate the impact of α1C palmitoylation on Ca(v)1.2 function. The function of the channel was assessed by measuring Ca^2+^ transients in adenovirus-infected human Induced Pluripotent Stem Cell (iPSC)-derived CMs in the presence of 1 µM of the dihydropyridine nifedipine to block the native channel. The commercially sourced iPSC-derived CMs displayed spontaneous contractile activity and are “embryonic” in their electrophysiology and excitation–contraction coupling process with <10% of the Ca^2+^ transient derived from the sarcoplasmic reticulum (28). As such, both the rate of rise and the amplitude of the Ca^2+^ transient are entirely controlled by Ca(v)1.2, and the Ca^2+^ transient decay is controlled by both Ca(v)1.2 inactivation and Ca^2+^ efflux through the sarcolemmal Na/Ca exchanger NCX1. Identical quantities of wild-type and unpalmitoylatable α1C trafficked to the surface membrane in these cells (Fig. 7*A*). Although time to peak of the Ca^2+^ transient was not influenced by α1C palmitoylation, we recorded significantly smaller Ca^2+^ transients in cells expressing unpalmitoylatable α1C (Fig. 7 *B* and *C*). Ca^2+^ transients were significantly longer in cells expressing unpalmitoylatable α1C compared to WT, presumably due to the reduced Ca^2+^ entry leading to less CDI (Fig. 7 *C*–*E*).

To investigate the links between the shifts in Ca^2+^ transient amplitude and the Ca(v)1.2 activation mediated by palmitoylation, we used a computational model of iPSC-CM electrophysiology and Ca^2+^ handling. (29) As shown in Fig. 7*F*, shifting the Ca(v)1.2 activation parameter from the normal value (V50 = −10 mV) more positive by 10 mV to V50 = 0 mV reduces the action potential duration and the corresponding Ca^2+^ transient amplitude. Further shifts in V50 to +10 mV cause further decreases in Ca^2+^ transient amplitude. This simulation of the specific effect of decreased palmitoylation on Ca(v)1.2 alone reproduces the decreased Ca^2+^ transient observed experimentally.

### Palmitoylation Site Conservation in Ca(v) Isoforms.

**Fig. 8.**
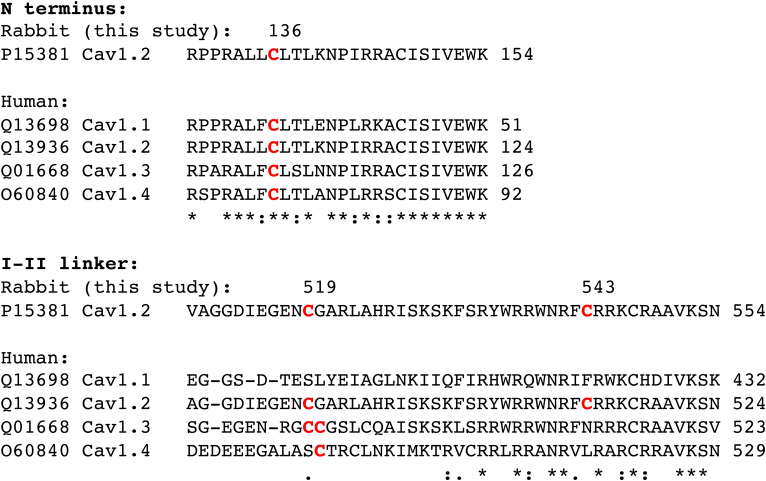
Palmitoylation site conservation in human Ca(v)1 channel isoforms. For clarity, regions of the rabbit α1C splice variant of Ca(v)1.2 CACH2A (UniProt accession number P15381) with palmitoylated cysteines identified in this investigation numbered and highlighted in red are shown above the corresponding regions of the human channels. Numbers at the end of each sequence are the numbering of the final amino acid in the region of each Ca(v)1 isoform shown. Human Ca(v)1 isoforms (UniProt accession numbers shown) were aligned using Clustal Ω. “*” below an amino acid indicates 100% conservation between isoforms; “:” indicates amino acids of highly similar properties; “.” indicates amino acids of weakly similar properties. The palmitoylation site in the Ca(v)1.2 N terminus is conserved in all isoforms. Ca(v)1.1 does not possess a cysteine analogous to C519 in the I–II linker, but Ca(v)1.3 and 1.4 do. C543 is unique to Ca(v)1.2.

An alignment of human Ca(v)1 isoforms is presented in Fig. 8. All retain the palmitoylation site in the N-terminal region. However, Ca(v) 1.1 completely lacks analogous palmitoylation sites in the I–II linker, while Ca(v) 1.3 and 1.4 possess a site analogous to Ca(v)1.2 C519 but not C543.

## Discussion

In this study, we set out to map palmitoylation sites in the pore-forming α1C subunit of the cardiac L-type Ca^2+^ channel Ca(v)1.2 and to define the effect that the palmitoylation of this subunit has on the channel’s function. Our results show that two regions of the protein are palmitoylated and that the voltage dependence of a channel formed from unpalmitoylatable α1C is significantly altered. This rightward shift of the Ca(v)1.2 voltage activation curve is consistent with reduced Ca^2+^ entry in iPSC-derived CMs, reducing the amplitude of the Ca^2+^ transient while simultaneously prolonging the transients because the extent of CDI is reduced.

### Events at the Membrane–Cytosol Interface Regulate Ca(v)1.2.

Two of the three palmitoylation sites in Ca(v)1.2, C136 and C543, lie in cytosolic regions of the channel that are positioned close to the membrane (27). While palmitoylation here might influence the TM domains, it is unlikely to significantly alter the local protein structure (and therefore function) since these regions are already close to the membrane. The third palmitoylation site, C519, which lies midway through the I–II linker, is considerably more distant from the TM domains. This region of the channel (for which structural information has not been resolved) is flanked by the α-interaction domain [AID, residues 458 to 475 in the rabbit splice variant used in this investigation, a highly conserved motif, to which the Ca(v)1.2 β subunit binds (3)] and by the first membrane-spanning segment of domain II. The I–II linker is a key regulator of channel gating both through its interaction with the β subunit (3) and through direct interactions with the plasma membrane (30). Single amino acid changes in this region of α1C profoundly alter Ca(v)1.2 behavior, causing Brugada (31) and Timothy (32, 33) syndromes, which are characterized by the shortening and prolongation of the electrocardiogram QT interval, respectively. Indeed, a loss-of-function mutation that causes Brugada syndrome [G490R (31) in human α1C] is immediately adjacent to C519 in the rabbit splice variant CACH2A used in this study. Given the importance of the I–II linker for channel function, future investigations will focus on the regulatory contribution of palmitoylation at C519.

The importance of the β subunit in regulating the trafficking and biophysical properties of L-type Ca^2+^ channels has long been established. Although each β variant has subtly different effects on channel function, the general principle of β association shifting the voltage dependence of activation and modifying inactivation properties holds for all β subunits investigated. An α-helix between transmembrane segment 6 of domain I and the AID is of particular importance for the functional effects of β. The rigidity of this helix, for example, is central to the ability of β to regulate channel inactivation (34). In this investigation, the rightward shift in the current–voltage relationship of Ca(v)1.2 formed from nonpalmitoylated α1C suggests that the usual leftward shift in this relationship induced by the presence of β has been blunted. We suggest this implies that the flexibility and/or structure of the region on the C-terminal side of AID (between AID and the first transmembrane domain of α1C repeat II) is also an important determinant of β’s influence on Ca(v)1.2 activation properties. It is inevitable that the flexibility of the I–II linker on one side of AID will influence the ability of β to move/restructure the other side since both regions are connected to β through their connection to AID. This investigation focused on the functional impact of α1C palmitoylation in the presence of β2a. An important question for future investigations will be whether the functional effects of α1C palmitoylation are different in the presence of different beta subunits.

The C-terminal end of the I–II linker in Ca(v)1.2 forms an α-helix that is amphipathic in character, with one face consisting almost entirely of basic amino acids and the other rich in aromatic residues (30). This region of Ca(v)1.2 binds PIP2 and is released from the membrane by phospholipase C activation, leading to changes in channel gating and run-down behavior (30). The palmitoylation of C543 within this helix would ensure continued membrane engagement in the absence of PIP2, potentially rendering Ca(v)1.2 insensitive to phosphoinositide breakdown. The palmitoylation of Ca(v) channel β subunits is known to change their sensitivity to PIP2, (35) and the relationship between PIP2 and both NCX1 (14, 16) and ATP-sensitive potassium channels (36) is also modified by palmitoylation, suggesting a common theme in ion channel and transporter regulation.

### Ca(v)1.2 Biophysical Properties in Health and Disease.

We modeled the impact of α1C palmitoylation on Ca^2+^ handling and action potentials of iPSCs, whose Ca^2+^ handling pathways are relatively immature. By controlling the duration of the cardiac action potential in adult CMs, the biophysical properties of Ca(v)1.2 directly influence the QT interval (9, 10, 37, 38)—a critical determinant of cardiac electrical stability. Our modeling data are therefore consistent with a role for α1C palmitoylation in controlling the QT interval in vivo. In the setting of cardiac arrhythmias, early after-depolarizations (EADs, transient depolarizations late in the cardiac action potential) are induced by activation of Ca(v)1.2 at voltages where the channel’s activation and inactivation curves overlap (the so-called window current). (39) EADs trigger the lethal arrythmias ventricular tachycardia (VT) and ventricular fibrillation (VF), and EAD susceptibility is directly controlled by the biophysical properties of Ca(v)1.2. Even small positive shifts in the V50 of Ca(v)1.2 will suppress EADs in ventricular (40) and atrial (41) myocytes because the window current is reduced. Our findings therefore suggest that strategies specifically decreasing palmitoylation of α1C would improve cardiac electrical stability and reduce susceptibility to VT and VF. Compounds capable of specifically depalmitoylating individual protein targets are currently in development and show promise (42, 43).

### Palmitoylation Sites in Ca(v)1.2.

Palmitoylation sites are commonly found near juxtamembrane amphipathic α-helices (44, 45). The proximity of the active site of the palmitoylating zinc finger Asp-His-His-Cys domain containing (zDHHC) family of enzymes to the bilayer (46) means that cysteines that are targeted for palmitoylation must be close to this bilayer. C543 lies on the hydrophobic face of the amphipathic α-helix at the carboxyl end of the I–II linker, while C547 (which is not palmitoylated) is one turn of this helix further on at the interface of the hydrophilic and hydrophobic faces. C136 and C147 in the channel amino terminus are also in membrane-proximal α-helices. Since they will be in close proximity to the active site of a palmitoylating enzyme (when C136 and C543 become palmitoylated), the lack of palmitoylation at C147 and C547 is somewhat surprising. This implies greater specificity to the palmitoylation reaction than is suggested by the concept of “stochastic palmitoylation” proposed for integral membrane proteins. (47) Of note, a recent in vivo proximity biotinylation study of cardiac Ca(v)1.2 α1C subunit identified three zinc finger Asp-His-His-Cys domain palmitoyl acyl-transferase (zDHHC-PAT) enzymes in close proximity to this protein (11). Future investigations will explore a functional link between these enzymes and Ca(v)1.2 palmitoylation. The fact that C519 and C543 are palmitoylated in the absence of TM domains, while C136 requires the TM domains to be present, suggests that different zDHHC-PATs with different substrate specificities are responsible for palmitoylating these different sites. Palmitoylation of the different sites may also occur at different points in the secretory pathway.

### Palmitoylation Site Conservation in Ca(v) Isoforms.

All Ca(v)1 isoforms retain the N-terminal palmitoylation site, but the palmitoylation sites in the I–II linker are less well conserved and entirely absent from Ca(v)1.1. In terms of the wider Ca(v) family, none possesses palmitoylation sites analogous to Ca(v)1.2 in their I–II linkers, while only the T-type channels Ca(v)3.2 and Ca(v)3.3 have cysteines analogous to Ca(v)1.2 C136 in the N terminus. An in-depth proteomic characterization of palmitoylation sites in the mouse brain previously identified the N-terminal palmitoylation site in Ca(v) 1.2, 1.3, and 2.1 and multiple palmitoylation sites in Ca(v) 3.2 and 3.3 (48). This suggests that palmitoylation is likely to be broadly important for regulating Ca(v) channel function.

## Future Questions and Concluding Remarks

We highlight that when investigating the functional impact of palmitoylation, loss-of-function experiments (functional characterization of nonpalmitoylated mutants) cannot readily be complemented by gain-of-function experiments (functional characterization after specifically enhancing palmitoylation of a protein of interest). It is conceivable, but unlikely, that the mutations introduced into α1C in this investigation influence Ca(v)1.2 function independently of changes in palmitoylation. Technologies that allow researchers to specifically enhance palmitoylation of individual cysteines will be a valuable addition to the palmitoylation tool kit but are not currently widely available.

Although we have ruled out a requirement for α1C to be palmitoylated in order for it to localize to buoyant, caveolin-enriched membrane microdomains, we cannot rule out the possibility that palmitoylation has more subtle effects on α1C subcellular localization. In particular, palmitoylation might influence the localization of Ca(v)1.2 to t-tubules [which have a unique phospholipid composition (49)] and/or the functional coupling between Ca(v)1.2 and the ryanodine receptor in ventricular myocytes. This question cannot be addressed in iPSC-derived myocytes but will be the subject of future studies in adult ventricular cells.

We find that ~60% of α1C is palmitoylated in ventricular myocytes, meaning palmitoylated and nonpalmitoylated α1C must coexist in the heart. Our results thus support the existence of functionally different subpopulations of Ca(v)1.2 in cardiac muscle. Little is currently known about the cellular control of palmitoylating and depalmitoylating enzymes. Important future questions to address about regulation of Ca(v)1.2 by palmitoylation include how rapidly α1C palmitoylation turns over, on what timescale channel properties can be “tuned” by palmitoylation, and whether any remodeling of the α1C palmitoylation and depalmitoylation pathways contributes to the well-established ventricular action potential abnormalities observed in heart failure (50).

## Methods

### Ethics Statement.

This study utilized primary ventricular myocytes from mice, rat, and rabbits. All protocols involving animals were approved by the University of Glasgow Animal Welfare and Ethics Review Board. Rodent cardiac tissues were collected postmortem after killing animals using a method designated Schedule 1 by the Animals (Scientific Procedures) Act 1986. Rabbit hearts were excised from terminally anesthetized, heparin-treated animals under the authority of a project license granted by the UK Home Office.

### Human Cardiac Tissue.

Cardiac tissue was kindly provided by Dr Kenneth Campbell at the University of Kentucky. The tissue collection program was approved by the University of Kentucky's Institutional Review Board. Kentucky Organ Donor Affiliates obtained informed consent from the family of each donor to use organs for research if they were not suitable for transplant. All donors (four females aged 38 to 61 y and three males aged 26 to 75 y) were classified as healthy controls, and cause of death was not cardiac. All samples were from the left ventricular endocardium of hearts that were not suitable for transplant. The protocols for tissue collection, preservation, and storage are described in detail elsewhere (51).

### Mutagenesis and Cloning.

For clarity, the numbering convention for amino acids in Ca(v)1.2 used throughout this investigation refers to the rabbit splice variant CACH2A. A plasmid expressing the human HHT-1 splice variant of α1C was kindly provided by Professor Chris Peers, University of Leeds, UK. Regions of this cDNA that encode intracellular loops of the protein were subcloned into the vector pEYFP-C1 (Clontech).

Plasmids to express split α1C (CFP-[I-II]-N-intein and C-intein-[III-IV]-YFP based on the rabbit splice variant CACH2A) and β2a-CFP were kindly provided by Professor Stanley Colecraft, Columbia University. Adenoviruses that express these proteins were generated using the Clontech Adeno-X system and purified and titered using standard techniques. Point mutations were generated using the QuikChange II Site-Directed Mutagenesis Kit (Agilent).

### Cells.

Calcium-tolerant, adult rabbit ventricular myocytes were isolated from the left ventricular free wall of male New Zealand white rabbits (2.8 to 3.2 kg) and of male Wistar rats (250 to 300 g) following perfusion with collagenase in the Langendorff mode. HEK-293 cells were cultured using standard conditions and were transfected using Lipofectamine 2000.

We used the Flp-In System (Invitrogen) to generate cells stably expressing tetracycline-inducible wild-type and unpalmitoylatable (C136A/C519A/C543A) α1C. Briefly, the HHT-1 splice variant of human α1C was subcloned into pcDNA5-FRT/TO, which was cotransfected with pOG44 Flp-Recombinase (Invitrogen) into Flp-In 293 T-REx cells. Cells were selected and maintained in the presence of hygromycin and blasticidin according to the manufacturer’s instructions. Commercially sourced Cor.4U human iPSC-derived cardiomyocytes™ (NCardia, Netherlands) were cultured using Cor.4U maintenance medium™ (NCardia, Netherlands) using the manufacturer’s recommended density of 30,000 cells/well in 10 µg/mL bovine fibronectin plasma (F1141, Sigma-Aldrich, Merck, Germany)-coated, 96-well, glass-bottom plate (MatTek, USA). (52)

### Palmitoylation Assays.

We adapted our PEG-switch assay that replaces palmitate with a 5-kDa methoxy PEG (21) by using a refinement that improves the reaction efficiency by PEGylating separately from the thioester cleavage step (53, 54). Briefly, free protein thiols were alkylated with 100 mM maleimide (in the presence of 2.5% sodium dodecyl sulfate (SDS), 100 mM 4-(2-hydroxyethyl)-1-piperazineethanesulfonic acid (HEPES), and 1 mM ethylenediaminetetraacetic acid (EDTA), pH 7.5) for 4 h at 40 ˚C. Excess maleimide was removed using acetone precipitation, followed by extensive washing of the protein pellet with 70% acetone. Proteins were resolubilized (1% SDS, 100 mM HEPES, and 1 mM EDTA, pH 7.5), and thioester bonds cleaved using 200 mM neutral hydroxylamine for 1 h at 37 ˚C. Hydroxylamine was removed by desalting (Zeba spin column), and free cysteines were PEGylated with 2 mM 5K-PEG maleimide (Sigma) for 1 h at 37 ˚C.

Palmitoylated proteins were purified from whole-cell lysates using acyl-RAC. Free thiols were alkylated with methyl methanethiosulfonate and palmitoylated proteins captured using thiopropyl Sepharose in the presence of neutral hydroxylamine. (44)

### Western Blotting.

Western blotting employed chemiluminescent detection using ChemiDoc XRS (Bio-Rad) and Odyssey-FC (LI-COR) imaging systems. Band intensities were calculated using Image Lab (Bio-Rad) and Image Studio (LI-COR) software. Anti-Ca(v)1.2 α1C subunit antibodies raised in rabbit and guinea pig were obtained from Alomone Labs, antibodies against flotillin 2 and caveolin 3 from BD Biosciences, and anti-GFP antibodies from Abcam and Protein Tech.

### Coimmunoprecipitation.

Cells were lysed in 2 mg/mL decaethylene glycol monododecyl ether (C12E10) in PBS supplemented with a protease inhibitor cocktail (Merck). Insoluble material was removed by centrifuging at 17,500 g for 5 min at 4 ˚C. Fluorescent protein–tagged proteins were immunoprecipitated using magnetic anti-GFP beads (ChromoTek) according to the manufacturer's recommendations.

### Preparation of Surface Membrane Proteins.

Primary amines on surface membrane proteins were biotinylated using 1 mg/mL sulfo-NHS-SS-biotin in Dulbecco’s PBS and then purified using streptavidin Sepharose as described previously. (14)

### Whole-Cell Voltage Clamping.

Whole-cell patch clamp was used to record Ba^2+^ or Ca^2+^ currents at room temperature from cells stably expressing tetracycline-inducible α1C 24 to 48 h after transfection with β2a (4 µg) and CD8 (0.25 µg) using Lipofectamine 2000 (6 µL) in a 35-mm dish. Dynabeads coupled with an anti-human CD8 antibody (Invitrogen) were used to visually identify cells which were successfully transfected.

Membrane currents were recorded with an AxoClamp 2B amplifier (Axon Instruments, Foster City, CA, USA) and WinWCP (John Dempster, University of Strathclyde, Glasgow, UK) software. Pipettes were pulled from borosilicate glass capillaries and when filled with intracellular solution had resistances between 2 and 6 MΩ. Associated voltage errors during recording were minimized by bridge balancing prior to sealing and expected to be <5 mV; no subsequent series resistance compensation was applied. Recordings were conducted from a holding potential of −60 mV using 500-ms voltage pulses at 1 Hz. I–V curves were obtained at voltage pulses between −60 mV and 60 mV with 10-mV increments. The pipette solution contained 120 mM NMDG-Cl, 1 mM MgCl_2_, 5 mM EGTA, 4 mM Mg-ATP, and 42 mM HEPES (pH 7.3 adjusted with methane sulfonic acid). Bath solution contained 40 mM CaCl_2_ or 40 mM BaCl_2_, 1 mM MgCl_2_, and 105 mM Tris (pH 7.3 adjusted with methane sulfonic acid). We did not correct for liquid junction potentials which were −22.8 mV for Ca^2+^ recordings and −23.1 mV for barium recordings (calculated according to the stationary Nernst–Planck equation using LJPcalc software).

From each cell, I–V curves in the range −60 to 60 mV were fitted to the Boltzmann equation as follows:

I = Gmax(Vm-Vrev)/(1 + exp(-(Vm-V50)/Ka)).

Here, Gmax is the maximal conductance, Vm is the membrane voltage, Vrev is the reversal potential of the current, Ka is the slope factor, and V50 is the half-maximum activation voltage.

The values obtained for Gmax and Vrev were then used to calculate fractional conductance values for each cell at each Vm using the following equation: G/Gmax = I/(Gmax(Vm-Vrev)).

G–V curves were fitted to the form of the Boltzmann equation as follows:

G/Gmax =1/(1+exp(−(Vm−V50)/Ka)).

The inactivation phases of Ca^2+^ currents generated by voltage steps from −60 mV to 20 mV were fitted with a single exponential using the modified Levenberg–Marquardt least squares minimization algorithm in WinWCP. This yielded inactivation tau values for comparisons between wild-type and unpalmitoylatable Ca(v)1.2-mediated currents.

### Ca^2+^ Transients in iPSC-Derived Myocytes.

Monolayers of iPSC-derived cardiomyocytes infected with adenoviruses that express wild-type or unpalmitoylatable α1C were loaded with 4 µM Fura-4F/AM (F14175, Thermo Fisher Scientific) in serum-free medium for 25 min at 37 °C, 5% CO_2_, and 80% humidity. Spontaneous Ca^2+^ transients were recorded in serum-free conditions in the presence of 1 µM nifedipine in an incubated stage maintained at 37 °C, 5% CO_2_, and 80% humidity in a CellOPTIQ platform (Clyde Biosciences Ltd., UK). (52, 55) Uninfected cells were quiescent in the presence of nifedipine. Monolayers of iPSC-derived cardiomyocytes were excited in an alternate fashion using 340- and 380-nm wavelength LEDs for 20 ms at each wavelength. Emission at greater than 570 nm was sampled separately for 340-nm excitation (F340) and 380-nm (F380) excitation wavelenths (56). Ca^2+^ transients were assessed from the fluorescence ratio (F340/F380) and were subsequently analyzed using CellOPTIQ analysis software (Clyde Biosciences, Ltd.) (55).

### Modeling Ca^2+^ Transients and Action Potentials in iPSC-Derived CMs.

The Paci-2013 IPSC cardiomyocyte model (29) was used to model the effect of altering steady-state activation of the L-type Ca^2+^ channel. This was implemented in Myokit, a Python-based modeling integrated development environment. The initial model used gating kinetics derived from Ma et al. (57) Activation kinetics were altered by increasing the activation voltage in the d_infinity equation.

### Sequence Alignments.

Ca(v) sequences were obtained from UniProt and aligned using Clustal Ω.

### Statistical Analysis.

All data are presented as mean ± SEM. Quantitative differences between groups were assessed using a one-way ANOVA followed by appropriate post hoc tests using GraphPad Prism.

## Data Availability

The datasets generated and analyzed during the current study are available at 10.5525/gla.researchdata.1352 (58).
